# Metabolic Profiling Provides Unique Insights to Accumulation and Biosynthesis of Key Secondary Metabolites in Annual Pasture Legumes of Mediterranean Origin

**DOI:** 10.3390/metabo10070267

**Published:** 2020-06-28

**Authors:** Sajid Latif, Paul A. Weston, Russell A. Barrow, Saliya Gurusinghe, John W. Piltz, Leslie A. Weston

**Affiliations:** 1 Graham Center for Agricultural Innovation, Locked Bag 588, Wagga Wagga, NSW 2678, Australia; pweston@csu.edu.au (P.A.W.); rubarrow@csu.edu.au (R.A.B.); sgurusinghe@csu.edu.au (S.G.); leweston@csu.edu.au (L.A.W.); 2 School of Animal and Veterinary Sciences, Charles Sturt University, Wagga Wagga, NSW 2678, Australia; 3 School of Agriculture and Wine Sciences, Charles Sturt University, Wagga Wagga, NSW 2678, Australia; 4 Plus 3 Australia Pty Ltd., P.O. Box 4345, Hawker, ACT 2614, Australia; 5 New South Wales Department of Primary Industries, Wagga Wagga, NSW 2678, Australia; john.piltz@dpi.nsw.gov.au

**Keywords:** pasture legumes, phytoestrogens, flavonoids, coumestans, polyphenols, proanthocyanidins, metabolic profiling, biosynthesis

## Abstract

Annual legumes from the Mediterranean region are receiving attention in Australia as alternatives to traditional pasture species. The current study employed novel metabolic profiling approaches to quantify key secondary metabolites including phytoestrogens to better understand their biosynthetic regulation in a range of field-grown annual pasture legumes. In addition, total polyphenol and proanthocyanidins were quantified using Folin–Ciocalteu and vanillin assays, respectively. Metabolic profiling coupled with biochemical assay results demonstrated marked differences in the abundance of coumestans, flavonoids, polyphenols, and proanthocyanidins in annual pasture legume species. Genetically related pasture legumes segregated similarly from a chemotaxonomic perspective. A strong and positive association was observed between the concentration of phytoestrogens and upregulation of the flavonoid biosynthetic pathway in annual pasture legumes. Our findings suggest that evolutionary differences in metabolic dynamics and biosynthetic regulation of secondary metabolites have logically occurred over time in various species of annual pasture legumes resulting in enhanced plant defense.

## 1. Introduction

Broadacre farming frequently occurs with livestock production throughout southeastern Australia, with the pasture phase of crop rotation sustaining both lamb and cattle enterprises [1]. Lamb and beef production account for the majority of livestock-related income in southeastern Australia (AU$22 billion in 2017) and global demand is projected to dramatically increase over the next decade (Australian Bureau of Statistics, 2018). Legumes are integral to livestock pasture production systems through provision of high quality forage for grazing livestock. Establishment of pasture species that are non-toxic, persistent, and high in nutritional quality is therefore critical for continued improvement of livestock productivity.

Traditionally, subterranean clover (*Trifolium subterraneum* L.) and lucerne (*Medicago sativa* L.) are the most widely utilized pasture species in prime lamb, wool, and cattle producing regions across southeastern Australia. Subterranean clover is compatible across various soil types and is tolerant of pH extremes [2,3]. Lucerne is a deep-rooted perennial species frequently established across diverse rainfall regions and is suitable for neutral or mildly alkaline soils. Recently the establishment of both pasture species has proven challenging to sustain livestock production due to a range of biotic and abiotic factors. For example, lucerne, which contains high protein content, has been shown to undergo rapid fermentation in the rumen resulting in increased incidence of bloat and potential loss of nitrogen due to excretion [4]. In addition, ingestion of lucerne and subterranean clover is associated with metabolic disorders including acute inflammation of both the small and large intestine (red gut), sodium deficiency and pregnancy-related toxemia [5,6,7].

Increased risk of metabolic disorders, the significant cost of establishment, and low persistence of traditional legume pastures in low-rainfall regions [8,9] has led to the introduction of novel annual pasture legume species originating from Mediterranean regions of Europe and northern Africa to Australia [3]. These include *Biserrula pelecinus* L. (biserrula), *Ornithopus sativus* Brot. (French serradella), *Ornithopus compressus* L. (yellow serradella), *Trifolium glanduliferum* Boiss. (gland clover), *Trifolium spumosum* L. (bladder clover), and *Trifolium vesiculosum* Savi. (arrowleaf clover). These accessions are characterized by their adaptation to deep, acid and sandy soils, drought tolerance, weed suppressive potential, and prolonged availability as feed for livestock [10,11]. While the nutritive value of traditional pasture legumes has been studied in southeastern and Western Australia, a detailed investigation of the phenolic chemistry of annual legumes has not been performed with respect to livestock production [2].

Legumes have evolved chemical defense mechanisms mediated by secondary metabolites, including phytoestrogens, to deter herbivores and plant pests [12,13]. In terms of phenolic chemistry and key secondary metabolites in pasture legumes, flavonoids represent a distinct class of secondary products with both positive and negative impacts on plant–microbial and plant–livestock interactions [14,15]. In general, they are classified by their chemical structure into subgroups including anthocyanidins and anthoxanthins (flavanones, flavans, and flavanonols) [16].

Significant levels of phytoestrogens are produced in pasture legumes including lucerne as well as various clover species and when present at significant levels can seriously reduce reproductive efficiency and livestock fertility [15,17,18]. Coumestans and isoflavone phytoestrogens are stable, non-steroidal secondary metabolites that mimic mammalian estrogen, an endogenous female sex hormone [15,19]. The affinity of these phytoestrogens in binding estrogen receptor-*β* can result in reproductive abnormalities during embryo development, and infertility in both sexes of grazing livestock [20]. Isoflavone phytoestrogens are typically stored either as glycosides or aglycones in pasture legumes [15,21].

**Figure 1:**
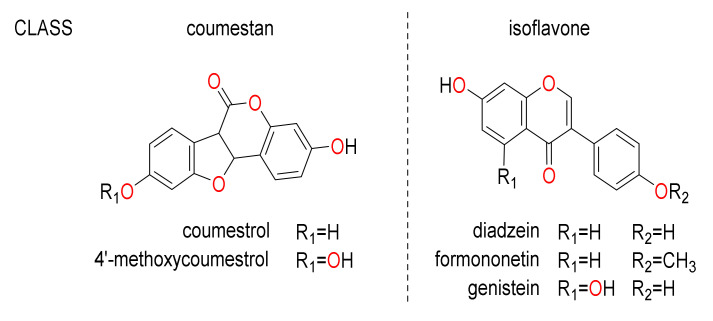
Phytoestrogens found in abundance in lucerne (*Medicago sativa*) and subterranean clover (*Trifolium subterraneum*).

Coumestans are non-flavonoid phytoestrogens, and include coumestrol and 4′-methoxycoumestrol, first isolated from white clover (*Trifolium repens* L.) and lucerne (*Medicago sativa* L.) in 1957 [22]. These polycylic aromatic metabolites are closely related biosynthetically to flavonoids (Figure 1). Elevated concentrations of phytoestrogens, including isoflavones, coumestrol, and related metabolites, from ingestion of fodder or feedstock have been implicated in estrogenic clinical signs in livestock as edematous vaginal and cervical tissue, hypertrophy of mammary glands, and milky secretions from elongated teats. Phytoestrogens in forage may also cause adverse effects in ovarian function resulting in loss of fecundity or early embryonic death [23]. Livestock grazing various *Trifolium* species exhibit varying tolerance to coumestans, which typically range in concentration from 25 to 200 mg kg^−1^ dry matter (DM) [15]. The presence of coumestrol at concentrations greater than ≈40 mg kg^−1^ DM in plant tissues is associated with reproductive inefficiency in sheep and cattle through disruption of several endocrine mechanisms [24,25].

Isoflavonoids are 3-phenylchromen-4-ones (3-phenyl-1,4-benzopyrone) and are important in regulating numerous interactions in higher plants. The isoflavone biosynthetic pathway is one of the most well elaborated pathways in plant secondary metabolism due to the importance of isoflavones as chemoattractants for rhizobia and their involvement in plant defense. Isoflavones are mainly derived from the phenylpropanoid pathway but can be generated through multiple pathways in many plant species [26]. Legumes possess a unique enzyme, isoflavone synthase (IFS), which is a cytochrome P450 monooxygenase that catalyzes the 2, 3 migration of the B-ring of naringenin or liquiritigenin, resulting in the production of various biologically active isoflavonoids [27]. The genes encoding enzymes in this pathway are tissue specific and are regulated both spatially and temporally in legumes [28]. Such catalytic enzymes are induced by various stress factors influencing plant condition, including climate, temperature, soil moisture availability, nutrient deficiency, and herbivory [29].

Polyphenolic compounds, including condensed tannins (proanthocyanidins), are another key group of metabolites possessing a range of biological and nutritional properties in grazing livestock. These compounds vary with regard to chemical structure, plant source, and target animal species [30]. For example, monomeric phenolic acids in forages are associated with enhanced milk production and acid-base imbalance in the rumen following microbial degradation [31]. Traditionally, the proanthocyanidins, which are oligomeric polyphenols, have been considered as anti-nutritional factors leading to reduced palatability of forages, but recent research has shown they provide several potential advantages to grazing livestock, including reduced risk of bloat at moderate intake of 3–4% of dry matter (DM) [32] and increased weight gain, while reducing greenhouse gas emissions [33,34,35] and parasite burden [36,37]. However, feed composition, age and physiological status of the animal are some of the key factors need to be taken into account while studying the effects of plant secondary metabolites on livestock [38,39].

There is a marked lack of information on the phytochemical profiles of aerial tissues of annual pasture legumes, particularly those recently introduced to the southern hemisphere from the Mediterranean. Recent advances in quadrupole time-of-flight mass spectrometry (MS-QToF) instrumentation have resulted in increasing usage of time-of-flight mass spectrometry (MS-ToF) instruments as quantitation tools as well as prediction of molecular formulae because of their high resolving power and wide dynamic range [40,41]. To broaden our understanding of the secondary chemistry of novel pasture legumes in Australia, we took a metabolomics approach using LC-MS-QToF to investigate (a) the distribution of secondary metabolites that may impact livestock performance, including flavonoids and phytoestrogens (coumestrol, 4′-methoxycoumestrol, formononetin, genistein and daidzein), in above-ground tissues of selected traditional and newly introduced annual pasture legumes grown under field conditions in southern Australia; (b) the total polyphenol and proanthocyanidin content to better delimit their prevalence in these pasture species, and (c) the biosynthetic pathways associated with the production of flavonoids and certain phytoestrogens in these plants.

## 2. Results

### 2.1. Quantification of Phytoestrogens

Concentration of coumestans in foliar tissues of all species ranged between 0.13 and 48.4 mg kg^−1^ and varied significantly across pasture species (Table 1). Both coumestrol and 4′-methoxycoumestrol accumulated at higher concentrations in leaf and stem compared to inflorescence tissues. Bladder clover possessed significantly higher concentrations of both coumestrol and 4′-methoxycoumestrol in leaf tissue compared to other pasture legumes (48.4 and 24.8 mg kg^−1^, respectively) while in stem tissue, bladder clover had the highest concentration of coumestrol (39.6 mg kg^−1^). Lucerne contained the highest concentration of 4′-methoxycoumestrol (27.7 mg kg^−1^) in any tissue of all species, and lucerne inflorescence tissue contained the highest concentrations of coumestrol and 4′-methoxycoumestrol (0.5 and 0.2 mg kg^−1^, respectively) of all annual pasture species examined.

Three isoflavones—daidzein, formononetin and genistein—are commonly found in pasture legumes and were also profiled in this study (Table 2). Isoflavone content in leaf (113.3–443.9 mg kg^−1^), stem (37.5–968.1 mg kg^−1^), and inflorescence (29.8–614.1 mg kg^−1^) varied significantly by both species and tissue (Table 2). Gland clover had the highest concentration of total isoflavones in leaf tissue (443.9 mg kg^−1^) while bladder clover had the highest concentration in stem and inflorescence tissue (968.1 and 604.1 mg kg^−1^, respectively). In terms of individual phytoestrogenic isoflavones in aerial tissues, formononetin concentration was greatest overall, followed by genistein at physiological maturity (Table 2). The production of phytoestrogenic isoflavones was greatest in clover species; specifically, daidzein concentration was highest in leaf tissues of gland clover at 120.2 mg kg^−1^. Production of these compounds in stems was greatest in bladder clover and subterranean clover (112.0 and 107.8 mg kg^−1^, respectively) when compared to other species. Subterranean clover contained the highest levels of daidzein (128.9 mg kg^−1^) in inflorescence tissue when compared to other annual pasture species.

Formononetin concentration in leaf tissue was significantly higher in the perennial legume lucerne (329.4 mg kg^−1^) than the other species, while for stem tissue, formononetin concentration was greatest in bladder clover (829.8 mg kg^−1^). Interestingly, genistein levels were greatest in all three tissues types in bladder clover compared to other species.

### 2.2. Effect of Biserrula Cultivar and Growth Stage on Phytoestrogen Levels

**Figure 2:**
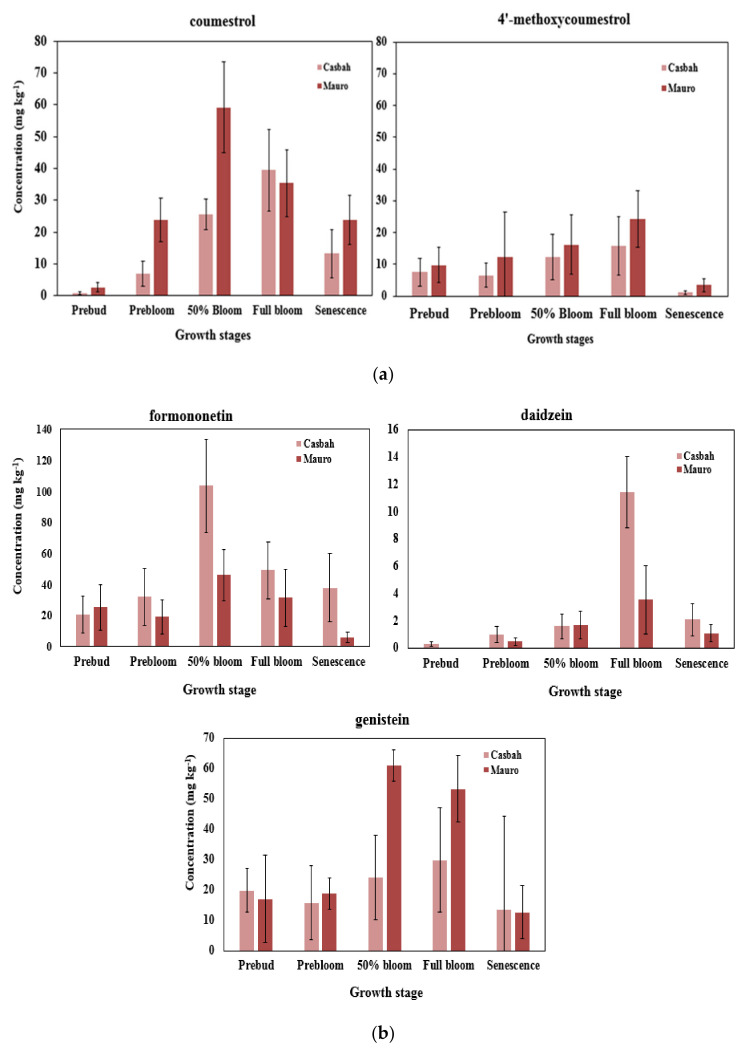
(**a**) The concentration of coumestrol and 4′-methoxycoumestrol at five growth stages i.e., pre bud, pre bloom, 50% bloom, full bloom, and senescence of field-grown biserrula cv. Casbah and cv. Mauro averaged over two years. Error bars indicate standard deviation; (**b**) the concentration of selected isoflavones at five growth stages i.e., pre bud, pre bloom, 50% bloom, full bloom, and senescence in biserrula cv. Casbah and cv. Mauro samples averaged over two years. Error bars indicate standard deviation.

Given the strong potential of biserrula to produce large quantities of biomass and suppress weeds successfully over time, further evaluation was performed to examine temporal effects on the accumulation of phytoestrogens in aerial tissues in both of the commercially available cultivars of biserrula in Australia. Analysis of tissues of Casbah and Mauro analyzed at five different growth stages showed that total concentrations of the phytoestrogens differed significantly between cultivars (Figure 2a,b). Phytoestrogen concentrations reached their maxima at either 50% bloom or full bloom while the lowest concentrations were observed at crop senescence. Significant differences in the concentration of coumestans between the two cultivars were limited to coumestrol at pre-bloom and 50% bloom stages; cv. Mauro was observed to produce greater levels of coumestans than cv. Casbah. The phytoestrogenic isoflavones formononetin and daidzein were more abundant in tissues of Casbah than Mauro, while the opposite was true for genistein.

### 2.3. Quantification of Total Polyphenols and Proanthocyanidins

Extractable TPC (total polyphenol content) ranged from 4.40 to 13.84 GAE and TPAC (total proanthocyanidins) ranged from 1.73 to 6.49 mg 100^−1^ g CE (Table 3). Gland clover contained significantly higher extractable TPC levels (13.84 mg 100^−1^ g) compared to all other pasture species. Extractable and bound TPAC was significantly higher in biserrula cv. Casbah compared to other species, while bound TPAC was only detected in the two biserrula cultivars (Casbah and Mauro) and the perennial legume, lucerne.

### 2.4. Abundance of Flavonoids and Their Glycosides

**Figure 3:**
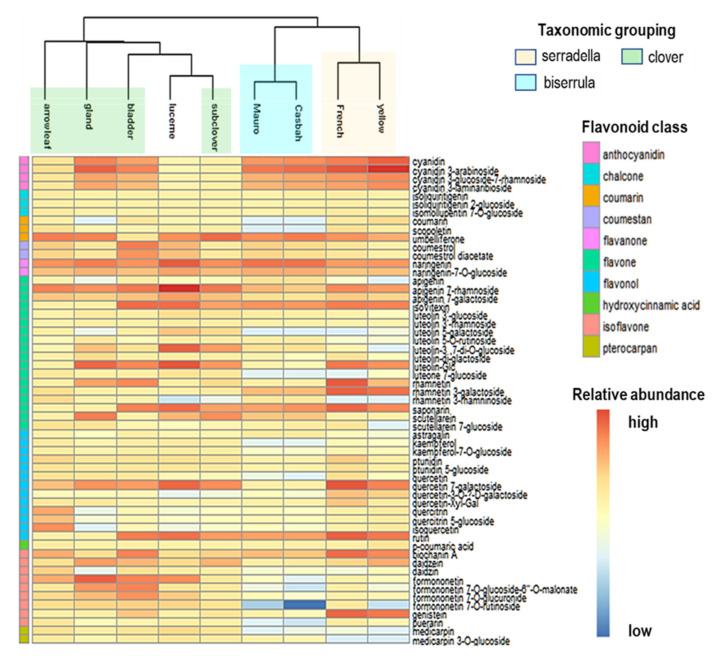
Hierarchal clustering of relative abundance of flavonoids, their glycosides, and coumestrol in leaf tissue in pasture legumes collected in 2016. Hierarchical clustering algorithm and Euclidean distance metric were used on normalized abundance using Mass Profiler Professional MPP (ver. 14.5 Agilent Santa Clara, CA, USA).

The clustering of molecular features profiled through non-targeted metabolic profiling revealed that molecular entities varied between species but were similar in species of the same genera (Supplementary Materials Figure S1). Over 5000 molecular features in total were successfully profiled in legume leaf tissues, with 1727 in stem and 1503 in inflorescence tissues (Figure S2). Interestingly, flavonoids and their glycosides accounted for the majority of constituents among all annotated major classes of secondary metabolites, based on verification with analytical standards as well as METLIN database comparisons. The relative abundance of various flavonoids and their glycosides in selected pasture legumes is presented in Figure 3. Interestingly, the total number of molecular features characterized in this study was highest in biserrula followed by French and yellow serradella, while gland clover exhibited the fewest total number of molecular features (Supplementary Materials Figure S1). However, gland clover, a recent introduction to Australia obtained from native pastures in the Mediterranean, exhibited a considerably higher abundance of secondary metabolites including flavonoids and their glycosides profiled in this study compared to other species (Figure 3). Both Casbah and Mauro cultivars of biserrula presented chemically similar profiles and exhibited relatively low abundance of flavonoids and their glycosides, in contrast to subterranean and gland clover.

## 3. Discussion

This study represents the first published report on quantification and distribution of secondary products, including flavonoids, phenolics, proanthocyanidins, and phytoestrogens, in aerial tissues of several annual pasture species (Table 1, Table 2, Table 3, Table 4 and Table 5, Figure 3). The metabolic profiling and metabolomics analyses employed in this study provided a comprehensive overview of the specific metabolites pertaining to the biosynthesis and regulation of flavonoids and related metabolites, particularly those with adverse impacts on grazing livestock. The accurate identification of secondary metabolites was facilitated by the use of quantitative liquid chromatography quadrupole time-of-flight mass spectrometry (LC-MS-QToF), allowing their detection and quantification at low concentrations (ng kg^−1^), with compounds identified by comparison with authentic standards or annotated using the METLIN library of secondary metabolites.

Along with various isoflavonoids, coumestans are produced in large quantities by members of the *Fabaceae*, commonly known as legumes, and most of these metabolites contribute to plant defense. Plant-produced coumestans are known to be associated with various biological activities, many of which can be attributed to their function as phytoestrogens and polyphenols [42]. 4- methoxycoumestrol has quantified using coumestan as surrogate standard [43,44]. Our findings suggest that the greatest concentration of coumestans occurs in leaf and stem tissues, in contrast to floral tissues which exhibited only trace quantities. This is in agreement with previous findings in traditional pasture legumes [45]. In addition, our results suggest that consumption of pure stands of novel annual pasture legumes such as biserrula, French serradella, yellow serradella, and arrowleaf clover at physiological maturity would likely pose no threat to herd fertility given the lower levels of coumestans than threshold suggested in the literature. In direct contrast, gland and bladder clover exhibited concentrations of coumestans at levels above the suggested tolerance limit of ≈40 mg kg^−1^ in leaf tissues at approximately 48 and 70 mg kg^−1^, respectively.

The most prevalent traditional pasture legumes in Australia, lucerne and subterranean clover, have been reported to consistently accumulate higher concentrations of coumestans at flowering in both leaf and stem tissues, but recurrent selection by plant breeders has recently resulted in reduced levels in many commercial cultivars. Our results indicated that both lucerne and subterranean clover cultivars selected in this study produced substantial concentrations of both metabolites, leading to a total concentration of 52 and 38 mg kg^−1^, respectively; levels which may exceed livestock tolerance limits (≈40 mg kg^−1^ DM). Considerably higher concentrations of coumestans in leaf and stem tissues of gland and bladder clover suggest that these cultivars have experienced relatively limited genetic improvement through breeding at this stage (Angelo Loi, personal communication), and produce significant levels of secondary products that may adversely impact livestock health. This observation further supports the hypothesis that coumestans play a role in plant defense against herbivory when established in natural settings [46]. Our findings also imply that these cultivars should be avoided as sole sources of forage for grazing livestock.

Naturally occurring isoflavones found predominately in the *Fabaceae* have been well described, largely based on their pharmacological activities, including estrogenic effects, in humans and animals. The isoflavonoids profiled in the present study demonstrated clear intra-species variation as well as variation associated with tissue type. Other researchers have also noted some chemical variance within the genus *Trifolium* [47]. The ability of plants to regulate production of secondary metabolites in response to biotic and abiotic stressors, including climate change, has been well documented [48,49,50,51] and recent studies have shown that these abilities extend to production of flavonoids [12,13].

Qualitative and quantitative variation in flavonoids and associated phytoestrogens profiled in our study is an indicator of both species and tissue specific adaptations to the environment, resulting in modulation in the expression of associated genes [52]. Although recent pasture studies have suggested that isoflavones are highly abundant in *Medicago* and *Trifolium spp*. and are frequently highest before full-bloom [53], considerable variation in total concentration has been reported in mature *Trifolium pratense* pastures, with those surveyed ranging between 9000 and 27,000 mg kg^−1^ DM [48,54,55]. Variations in isoflavone concentration may have been due to environmentally induced responses, time of sampling, extraction efficiency and recovery, and previous analytical workflows employed to profile individual isoflavones [21]. We specifically attempted to address these issues noted in previous published works by conducting experimentation in uniform and replicated field sites, creating composite replicated samples for each pasture plot, and performing sampling at similar stages of physiological maturity in both 2016 and 2017 growing seasons. In addition, we utilized an automated high-pressure extraction device to rapidly and uniformly extract all samples, thereby performing uniform extraction across treatments and replicates, and reducing the possibility of plant to plant variation or inefficiencies associated with manual extraction protocols.

Several phytoestrogenic flavonoids, such as formononetin, the most abundant isoflavone detected followed by and genistein, were prevalent at physiological maturity (50% flowering) in all pasture legumes. These observations are in agreement with previous reports pertaining to various *Trifolium* species [48,56,57,58]. Recent findings also suggested a greater abundance of phytoestrogens and isoflavonoids in leaf tissues when compared to stems and inflorescence [53]. Biosynthesis and subsequent distribution of isoflavones among leaf, stem, and floral tissues was impacted by cultivar, physiological growth stage, and climatic conditions under which plants were maintained [59]. In *Trifolium pratense* (perennial red clover), isoflavone concentrations were also found to be impacted by growth stage, with inflorescence tissue containing equivalent concentrations of isoflavones to those in leaf tissue in initial bloom stages, with subsequent declines in floral tissue as the crop entered full bloom stage [48]. The disproportionately high levels of formononetin in bladder clover observed in our study suggests that modulation of the phenylpropanoid pathway may, in fact, be species specific and is important in determination of terminal isoflavone accumulation in the pathway.

Only arrowleaf clover, gland clover, bladder clover, and subterranean clover exhibited total isoflavone concentrations above the livestock-safe threshold level of ≈280 mg kg^−1^ DM (Table 4) and therefore, all four of these *Trifolium* spp., could theoretically adversely impact the reproductive performance of livestock. Hashem et al., 2016 similarly reported that cattle fed solely on *Trifolium alexandrinum* (berseem clover) containing isoflavones at high concentrations of ≈280 kg^−1^ DM exhibited hormonal disruption and reduced fertility, comparable to similar cattle grazing other species [18]. Interestingly, lambs grazing *Trifolium pratense* with high levels of isoflavones exhibited weight gain compared to cultivars low in isoflavones, but macromolecular interactions impacting plant nutrition and subsequent weight gain were not fully explored in that study [60]. Future studies investigating the production of phytoestrogens, including coumestans and isoflavones at various growth stages will be important to optimize seasonal grazing.

**Figure 4:**
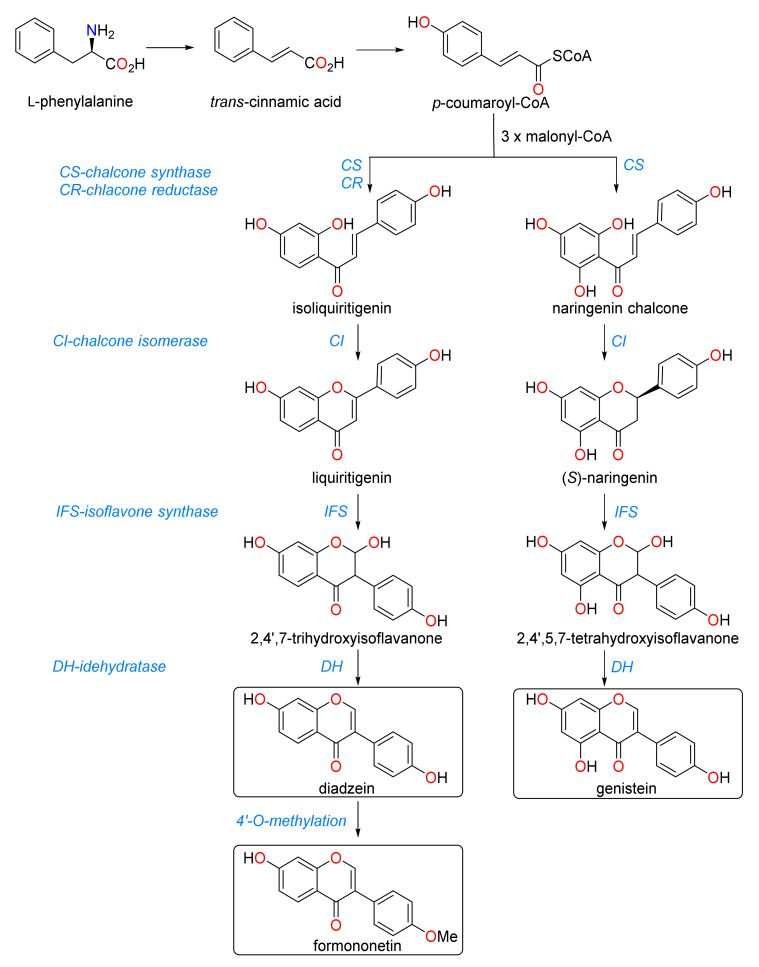
Biosynthetic pathway leading to the formation of various isoflavones including phytoestrogenic isoflavones (boxed) [29,64].

Isoflavones are synthesized as part of the phenylpropanoid pathway (Figure 4), which has multiple branches common to both legumes and non-legumes. The phenylpropanoid pathway leads to the generation of lignins, anthocyanins, phytoalexins and flavonoids, including isoflavones, as a means of plant protection against stress or predation [29]. Encoding enzymes in this pathway are both developmentally and tissue-specifically regulated, and environmental stressors such as exposure to UV light, drought, prolonged cold, pathogen attack, and nutrient deficiency may also influence end products. Isoflavone synthase (IFS) is a key enzyme involved in the production of an array of isoflavones from naringenin, a common phenylpropanoid pathway intermediate [27]. Legumes can produce both daidzein from the intermediate compound liquiritigenin, and genistein from the intermediate naringenin chalcone in alternative branches of the phenylpropanoid pathway (Figure 4). The presence of multiple copies of enzymes such as chalcone synthase and IFS in species within the *Fabaceae* allows for the differential regulation of isoflavone biosynthesis in response to both developmental and environmental stimuli [61,62].

The flavonoid precursors and various isoflavones profiled in this study varied qualitatively and quantitatively among the annual pasture species investigated, suggesting that these species differ with respect to their metabolic dynamics and ability to regulate flavonoid biosynthesis. Daidzein and formononetin are produced from one branch of the phenylpropanoid pathway while genistein is produced from another branch. Both branches originate from *p*-coumaroyl-CoA, but the bias of the pathway towards one branch or the other is typically determined by the equilibrium between the enzymes chalcone synthase (CS) and chalcone reductase (CR) [27]. Greater production of formononetin in the pasture legume extracts profiled in this study, in contrast to the other isoflavones (daidzein and genistein) (Table 2), suggests that flavonoid biosynthesis in some pasture legumes is biased towards the branch terminating in production of formononetin as opposed to the alternate pathway ending in genistein. These findings are also consistent with previous observations [27,63]. Of note, close metabolite clustering and a higher concentration of genistein in both cultivars of biserrula and serradella species (as opposed to the more typical daidzein and formononetin) suggested potential overexpression of the gene encoding CS for the conversion of *p*-coumaroyl-CoA to naringenin chalcone in biserrula and serradella (Figure 4). This observation is consistent with recent results of a study investigating isoflavone profiles in food-related species in the *Fabaceae* family where higher genistein concentrations discriminated the genera *Biserrula* and *Ornithopus* from other members of the family [52].

Isoflavones tend to accumulate at highest concentrations at physiological maturity of the plant in *Trifolium* spp. as they commence and complete flowering [65]. To assess the impact of growth stage on the abundance of key phytoestrogens, we further profiled phytoestrogens in two common biserrula cultivars, first commercialized in Australia in 2001, at five different growth stages. Interestingly, we observed no significant differences in coumestan concentrations between the two cultivars, despite the fact that the cultivars were initially isolated from geographically separate locations in the Mediterranean. This suggests that the phenylproponoid pathway was highly conserved in this species, particularly in early growth stages, and was not affected by genotypic differences. However, the production of phytoestrogenic isoflavones varied significantly at 50% bloom and full bloom stages (Figure 2a,b). A previous study also described a significant difference in the accumulation of isoflavonoids (formononetin and biochanin A) in different cultivars of red clover, peaking at 50% bloom and full bloom stage [48]. Our results demonstrated that concentrations of all phytoestrogens assessed were lowest at senescence, and are in agreement with earlier observations in red clover [65]. As noted previously, the genes encoding enzymes for the biosynthesis of isoflavones are developmentally regulated and are frequently influenced by environmental stressors and various biotic factors [29,49,62], some of which are likely experienced in southeastern Australia during a typical growing season. Breeding programs in annual pasture legumes can therefore exploit the bias of the phenylpropanoid pathway by targeted manipulation of genes involved in the biosynthesis of genistein and daidzein through ectopic expression of specific transcription factors over various growth stages [66].

Pasture legume samples collected from multi-year field trials were also subjected to quantification of extractable and bound total polyphenol content (TPC) and total proanthocyanidin content (TPAC) (Table 3). The important role of TPAC in pasture legumes in reducing herbivory by impacting palatability attributes has been elucidated previously [46]. This is the first study to report on both TPC and TPAC in annual self-regenerating pasture legumes at physiological maturity and our findings support previous reports suggesting a similar range for other related pasture legumes [33,67,68]. The range of TPAC in *Ornithopus* reported in a previous study [32] was between 2 g 100 g^−1^ and 2.5 g 100 g^−1^, which is also in agreement with the current study. However, proanthocyanidins in *Onobrychis viciifolia* (sainfoin) were reported to be up to 10 times higher than measured in this study and this discrepancy could be associated with species, methodology, or growth stage of sampling [69]. Of note is that previous studies using similar assays did not detect measurable TPAC in lucerne [70,71]. This could be due to differences in cultivar genetics and growth conditions; however, we also observed that lucerne exhibited the lowest extractable TPC and TPAC levels of all legumes surveyed. A high concentration of extractable TPAC was noted in biserrula cv. Casbah; both cultivars also exhibited high levels of bound TPAC in their cell walls. High TPAC levels suggest the potential for biserrula to limit pathogen and herbivore attack or reduced palatability to grazing livestock [72], but may potentially offer a cost-effective opportunity for this species to be integrated into multi-species mixtures to reduce parasite burden, an outcome suggested by recent replicated trials with biserrula on grazing sheep. At this time, specific bioactive proanthocyanidins in biserrula remain unidentified.

This study employed metabolic profiling approaches for identification and quantification of a number of more common secondary plant metabolites, particularly flavonoids. Our results clearly demonstrate that (a) this collection of annual pasture legumes produces a diverse array of flavonoids and other phytochemicals associated with plant defense, and in some cases less desirable phytoestrogens or proanthocyanidins and (b) the regulation of the biosynthesis of flavonoids and related metabolites such as coumestans through the phenylpropanoid pathway occurs at multiple branch sites and as reported could be impacted by elicitation in response to various biotic factors including predation and herbivory. Plants have thus developed various evolutionary adaptations for making regulatory decisions to produce an array of secondary metabolites which can provide them with multiple ecological benefits. In some cases, a single compound or related family of compounds can exhibit multiple biological functions in plants [64,73]. Despite significant differences in biological functions of various plant metabolites which are frequently concentration dependent, related compounds often share common biosynthetic pathways while others, including flavonoids, can originate from diverse biosynthetic pathways and precursors [51,74]. Evolutionarily, this chemical diversity or flexibility provides higher plants with a cost-effective strategy for further resource allocation or reallocation. As an example, our results suggest that both lucerne and gland clover have the ability to upregulate the synthesis of formononetin from daidzein while bladder clover evolved to upregulate biosynthesis of genistein from 2′,4′,5,7 tetrahydroxy-isoflavone (Figure 4).

Our study results also support the hypothesis that high concentrations of key phytoestrogens, TPC and TPAC in annual pasture legumes are associated with flavonoid abundance. Interestingly, the relative abundance of total molecular features profiled through non-targeted metabolic profiling was highest in biserrula leaf tissues followed by serradella, while gland clover leaf tissue exhibited the fewest molecular features. In contrast, gland clover exhibited a higher concentration of flavonoids and their glycosides, suggesting that trade-offs in metabolite production and regulation occur in pasture legume species; i.e., if flavonoid synthesis is upregulated, then the expression of TPAC, TPC, and other polyphenols may be downregulated.

## 4. Materials and Methods

### 4.1. Chemicals

Analytical grade acetone, acetic acid, anhydrous sodium acetate, ethanol, hexane, hydrochloric acid, potassium chloride, sodium carbonate, and sulfuric acid were obtained from Chem Supply (Port Adelaide, South Australia, Australia). High pressure liquid chromatography (HPLC) grade methanol, acetonitrile, formic acid, and ammonium acetate as well as analytical standards (Folin–Ciocalteu reagent, vanillin, gallic acid, cyanidin-3-*O*-glucoside, (+)-catechin, coumestrol, formononetin, genistein, and naringenin) were purchased from Sigma Aldrich (Australia).

### 4.2. Plant Material

Monocultures of selected pasture legumes listed in Table 4 were established in 2016 and 2017 (Experiment 1) at the Charles Sturt University research farm in Wagga Wagga, NSW, Australia (35.04° S, 147.36° E) on a red sodosol soil [75]. Each planting was arranged as a randomized complete block design with five replications. Individual pasture legumes were established in plots by direct-seeding with a drill adapted for small-seeded legumes in late May to early June of each year, with plots measuring approximately 4 × 20 m. Following on from experimentation to assess legume establishment and performance [75], above-ground plant tissues (leaf, stem, and flower) were collected from established plots in the third week of October in 2016 and 2017 after >50% flowering was achieved in each species. This corresponded to the growth stage in which peak concentrations of phytoestrogen have been reported in vegetative tissue [76]. An additional trial was also established at Charles Sturt University research farm in 2016 and 2017 which focused on chemical composition in two newly released biserrula cultivars (cv. Casbah and Mauro) using four replicates per cultivar (Experiment 2). In this case, composite fresh plant samples were collected from each plot (two sub-samples per cultivar) at pre-bud, pre-bloom, 50% bloom, full bloom, and senescence between mid-July to mid-November in 2016 and 2017, approximately every three weeks. All plant material was placed on ice at collection and subsequently stored at −20 °C until extraction.

### 4.3. LC-QToF-MS Analysis

Metabolic profiling of plant tissues was performed using an Agilent 1290 Infinity LC system equipped with a quaternary pump, diode array detector (DAD), degasser, temperature-controlled column (25 °C), and cooled auto-sampler compartment (4 °C) which was coupled to an Agilent 6530 quadrupole time-of-flight (QToF) mass spectrometer (MS) with an Agilent Dual Jet Stream ionization source (Agilent Technologies, Melbourne, Australia). Full-scan mass spectra were acquired over an *m/z* range of 100–1700 Da at a rate of two spectra/second in both positive and negative ion modes. Chromatographic separation was achieved using a reverse-phase C_18_ Poroshell column (2.1 × 100 mm, 2.7 μm particle size) (Agilent Technologies, Santa Clara, CA, USA) equipped with a C_18_ guard column (2.1 × 12.5 mm, 5 μm particle size) (Agilent Technologies, CA, USA) using a flow rate of 0.3 mL min^−1^. The column was equilibrated for 40 min prior to analysis. Separation was obtained with a gradient of solvent A, [water (Milli-Q, TKA-GenPure, Germany) + 0.1% formic acid (LC-MS grade, LiChropur^®^, 98–100%, Sigma-Aldrich, MO, USA)] and solvent B [95% HPLC-grade acetonitrile (RCI Labscan, Bangkok, Thailand) + 0.1% formic acid]. The solvent gradient was as follows: 5% B for 0.5 min increasing to 100% B over the next 16.5 min, then holding at 100% B for 23 min and returning to 5% B from 23.1 min to 29 min. The DAD monitored absorbance across a range of wavelengths from 210 to 635 nm. Injection volume was 10 µL for each sample. Nitrogen was used as the drying gas at 250 °C and a flow rate of 9 L min^−1^. Five biological replicates for each treatment were analyzed. Phytoestrogens (isoflavones and coumestans) identified in annual pasture legumes in the current study are summarized in Table 5.

### 4.4. Extraction of Polyphenols

Foliar and inflorescence samples (1 g) from each pasture legume were freeze-dried and ground manually to a fine powder (≈1–2 mm size) using a mortar and pestle. Samples were extracted as described previously [77] with minor modifications. Briefly, ground foliar tissue was defatted with *n*-hexane, and residues were extracted three times with 20 mL of solvent (acetone:water:acetic acid 70:29.5:0.5 *v*/*v*/*v*). The three aliquots were combined, and solvent was removed using a rotary evaporator. The residue was lyophilized and reconstituted in 50% aqueous methanol to a final concentration of 1 g mL^−1^ for storage at −20 °C until required.

### 4.5. Quantification of Total Polyphenol Content

Total free phenolic content was determined as described previously [78] with minor alterations. Briefly, 125 µL of the extract was mixed with 125 µL of Folin–Ciocalteu reagent and 500 µL of deionized water and incubated in the dark for 6 min. The solution was neutralized by adding 1.5 mL 7% aqueous sodium carbonate solution and further incubated in the dark for 90 min, after which absorbance was measured at 725 nm using a UV/Vis spectrophotometer (FLUOstar Omega, BMG Labtech, Offenburg, Germany) against a methanol control. Total phenolic content was expressed as mg 100^−1^ g of gallic acid equivalent (GAE). The experiment was repeated twice with three technical replicates.

### 4.6. Quantification of Total Proanthocyanidin Content (TPAC)

TPAC was quantified using a vanillin assay as described previously [79,80]. Briefly, 0.2 mL of extract was added to 0.5 mL of 1% (*w*/*v*) vanillin in methanol and 0.5 mL of 25% sulfuric acid in methanol. The extract was thoroughly blended using a vortex mixer and placed in a water bath at 37 °C for 15 min. Absorbance was measured at room temperature at a wavelength of 500 nm. The total proanthocyanidin content was determined as mg 100^−1^ g of (+)-catechin equivalent (CE). The experiment was performed with three technical replicates and the experiment was repeated twice.

### 4.7. Statistical Analysis

A matrix of molecular features characterized by mass to charge ratio (*m/z*) and retention time (RT) was generated using MassHunter Workstation Qualitative software version B07.00, MassHunter Profinder (version B.08.00), Mass Profiler Professional (MPP version 14.5) and Personal Compound Database Library (PCDL) (Agilent Technologies, CA, USA). Molecular features were extracted and binned/aligned using parameters as follows: peak height ≥ 10,000 counts, compound ion count threshold of two or more ions, compound alignment tolerances 0.00% + 0.15 min for RT and 20.00 mg kg^−1^ ± 2.00 mDa for mass using Profinder. Molecular features which were present only in three samples out of five were included in the analysis. Compounds were tentatively identified by matching molecular entities with PCDL entries having similar accurate mass, RT, and mass spectra (generated from analytical standards) where possible and the METLIN metabolomics database (version B 07.00, Agilent Technologies, CA, USA) otherwise. All descriptive statistical analyses were performed using Statistix (STATISTIX software, version 4.1; Analytical Software, Tallahassee, FL, USA) and standard deviations were calculated and reported where possible.

## 5. Conclusions

In conclusion, this study has extended our understanding of secondary metabolism associated with the biosynthesis of phytoestrogens and proanthocyanidins in annual and perennial legumes. Metabolic profiling performed with a variety of hardseeded annual pasture legumes demonstrated that phytoestrogens quantified in this study were at concentrations insufficient to pose negative impact on livestock production with the exception of gland clover, bladder clover, and lucerne. Results of the study also suggest that annual hardseeded pasture legumes of Mediterranean origin offer viable and sustainable alternative pasture options for mixed farming systems of southeastern New South Wales. In the future, the use of an integrated experimental approach including multi-omics platforms could also potentially provide deeper insights into pathway dynamics and regulation of associated genes important in the production of secondary metabolites in pasture legumes. Our findings along with those of Butkut et al., 2018 suggest strong potential to improve legume-based forage quality through recurrent selection or engineering for species- or cultivar-specific phytochemicals [53]. In addition, optimization of livestock management to reduce health and reproductive issues by selective grazing through timing of animal movement, manipulation of plant growth stage at harvest, and appropriate selection of pasture species mixtures is also warranted.

## Figures and Tables

**Table 1:** The concentration of coumestrol and 4′-methoxycoumestrol (mg kg^−1^) at physiological maturity in selected annual pasture legume species.

Tissue Type	Pasture Species	Coumestrol	4′-Methoxycoumestrol	Total Coumestans
Leaf	Arrowleaf clover	14.7 ± 2.2	5.9 ± 1.5	20.6 ± 5.1
	Biserrula cv.* Casbah	13.1 ± 2.7	5.2 ± 1.7	18.2 ± 6.5
	Biserrula cv. Mauro	14.6 ± 2.4	7.4 ± 5.5	22.0 ± 4.2
	Bladder clover	48.4 ± 10.6	24.8 ± 17.7	73.2 ± 13.6
	French serradella	ND	ND	ND
	Gland clover	27.1 ± 11.1	21.1 ± 5.6	48.2 ± 3.5
	Lucerne	31.8 ± 14.4	20.2 ± 7.5	52.0 ± 6.7
	Subterranean clover	25.1 ± 8.6	12.5 ± 4.5	37.6 ± 7.3
	Yellow serradella	ND	ND	ND
	LSD (*p* < 0.05)	9.3	5.4	13.1
Stem	Arrowleaf clover	24.2 ± 5.3	6.1 ± 4.6	30.3 ± 10.5
	Biserrula cv. Casbah	ND	ND	ND
	Biserrula cv. Mauro	ND	ND	ND
	Bladder clover	39.6 ± 7.7	18.7 ± 3.6	58.3 ± 12.1
	French serradella	ND	ND	ND
	Gland clover	26.2 ± 14.4	3.6 ± 1.9	29.8 ± 13.0
	Lucerne	36.6 ± 16.5	27.7 ± 12.8	64.3 ± 5.1
	Subterranean clover	25.4 ± 7.5	7.1 ± 1.6	32.5 ± 10.6
	Yellow serradella	ND	ND	ND
	LSD (*p* < 0.05)	9.4	14.5	17.5
Inflorescence	Arrowleaf clover	ND	0.1 ± 0.1	<0.1
	Biserrula cv. Casbah	ND	ND	ND
	Biserrula cv. Mauro	ND	0.2 ± 0.1	<0.1
	Bladder clover	0.2 ± 0.2	<0.1	<0.1
	French serradella	ND	ND	ND
	Gland clover	0.1	ND	<0.1
	Lucerne	0.5 ± 0.1	0.2 ± 0.1	0.7 ± 0.1
	Subterranean clover	0.3 ± 0.1	ND	0.3 ± 0.1
	Yellow serradella	ND	ND	ND
	LSD (*p* < 0.05)	0.2	0.1	0.2

ND denotes compounds not detected. Least significant difference (LSD) differentiated between concentration means. Values represent the arithmetic mean of five replicates ± standard deviation. *cv. is acronym for cultivar.

**Table 2:** The concentration of daidzein, formononetin and genistein (mg kg^−1^) in annual pasture legume species at physiological maturity, post-flowering.

Tissue Type	Pasture Species	Daidzein	Formononetin	Genistein	Total
Leaf	Arrowleaf clover	55.1 ± 26.4	222.9 ± 84.4	28.3 ± 6.8	306.3 ± 94.3
	Biserrula cv. Casbah	11.5 ± 3.5	73.6 ± 51.3	116.8 ± 2.8	192.5 ± 18.7
	Biserrula cv. Mauro	6.1 ± 0.7	58.9 ± 17.3	124.7 ± 12.5	189.7 ± 24.0
	Bladder clover	95.0 ± 2.1	152.3 ± 18.9	184.1 ± 26.3	431.4 ± 32.8
	French serradella	47.7 ± 12.5	35.5 ± 48.6	130.1 ± 7.6	113.3 ± 8.1
	Gland clover	120.3 ± 16.1	226.1 ± 20.5	97.5 ± 10.8	443.9 ± 61.4
	Lucerne	48.2 ± 13.3	329.4 ± 192.7	61.5 ± 10.1	439.1 ± 141.9
	Subterranean clover	109.1 ± 14.8	157.6 ± 47.3	72.1 ± 244.3	318.8 ± 40.5
	Yellow serradella	89.0 ± 13.	91.6 ± 16.6	118.4 ± 5.4	219.1 ± 43.0
	LSD (*p* < 0.05)	24.9	37.5	34.8	---
Stem	Arrowleaf clover	9.8 ± 3.2	147.7 ± 58.2	27.6 ± 5.2	185.1 ± 67.1
	Biserrula cv. Casbah	7.5 ± 0.9	23.9 ± 12.2	6.1 ± 1.9	37.5 ± 8.9
	Biserrula cv. Mauro	5.1 ± 1.4	60.4 ± 18.4	9.9 ± 3.7	75.4 ± 27.4
	Bladder clover	112.0 ± 33.5	829.8 ± 144.9	126.1 ± 5.4	968.1 ± 394.7
	French serradella	75.2 ± 23.3	96.7 ± 34.4	104.1 ± 18.9	276.0 ± 13.4
	Gland clover	3.4 ± 0.5	239.5 ± 18.4	88.1 ± 37.4	331.0 ± 107.0
	Lucerne	7.5 ± 2.3	120.6 ± 35.3	15.9 ± 5.1	144.0 ± 56.4
	Subterranean clover	107.8 ± 18.9	36.2 ± 6.4	18.2 ± 8.3	162.2 ± 42.4
	Yellow serradella	42.5 ± 7.2	77.7 ± 14.2	20.3 ± 6.9	140.5 ± 25.9
	LSD (*p* < 0.05)	20.8	47.9	19.8	---
Inflorescence	Arrowleaf clover	1.3 ± 0.3	98.2 ± 24.5	18.4 ± 4.6	117.9 ± 45.3
	Biserrula cv. Casbah	2.2 ± 0.5	39.6 ± 9.9	10.2 ± 2.5	52.0 ± 17.6
	Biserrula cv. Mauro	1.7 ± 0.4	18.5 ± 4.6	9.6 ± 4.1	29.8 ± 7.5
	Bladder clover	19.3 ± 4.8	435.8 ± 108.9	158.9 ± 37.2	614.1 ± 190.6
	French serradella	10.5 ± 2.6	98.8 ± 24.7	19.9 ± 4.9	129.2 ± 43.4
	Gland clover	68.3 ± 17.1	184.8 ± 46.2	68.3 ± 17.1	321.0 ± 60.1
	Lucerne	25.5 ± 6.4	182.6 ± 45.6	25.7 ± 6.4	233.8 ± 81.1
	Subterranean clover	128.9 ± 32.2	187.6 ± 46.9	148.7 ± 39.8	465.2 ± 26.3
	Yellow serradella	14.6 ± 3.6	17.6 ± 4.4	25.8 ± 6.6	58.00 ± 5.2
	LSD (*p* < 0.05)	16.8	39.2	24.5	---

Least significant difference (LSD) was used to differentiate between concentration means. Values represent the arithmetic mean of five replicates ± standard deviations.

**Table 3:** Concentration of extractable and bound total polyphenol and total proanthocyanidins in foliar tissues of pasture legumes.

Pasture Species	TPC		TPAC	
	Extractable	Bound	Extractable	Bound
Arrowleaf clover	10.50^b^	0.24^de^	3.71^c^	ND
Biserrula cv. Casbah	7.69^cde^	0.43^cd^	6.49^a^	8.87^a^
Biserrula cv. Mauro	6.24^def^	0.11^e^	4.29^b^	4.06^b^
Bladder clover	6.67^def^	0.45^bc^	1.73^c^	ND
French serradella	8.29^bcd^	0.35^cd^	1.94^c^	ND
Gland clover	13.84^a^	0.57^bc^	2.12^c^	ND
Lucerne	4.40^f^	0.11^de^	2.05^c^	0.33^c^
Subterranean clover	9.39^bc^	0.62^bc^	4.52^ab^	ND
Yellow serradella	5.17^ef^	0.72^b^	1.94^c^	ND
LSD	2.62	0.28	2.16	1.95

Values represent arithmetic mean of five replicates. Values followed by the same superscript in each column are not significantly different (*p* < 0.05) as determined by ANOVA.

**Table 4:** Scientific, common, and cultivar names of annual pasture legumes evaluated in this study.

Scientific Name	Common Name	Cultivar
*Trifolium vesiculosum* Savi.	Arrowleaf clover	Cefalu
*Biserrula pelecinus* L.	Biserrula	Casbah, Mauro
*Trifolium spumosum* L.	Bladder clover	Bartolo
*Ornithopus sativus* Brot.	French serradella	Margarita
*Trifolium glanduliferum* Boiss.	Gland clover	Prima
*Trifolium subterraneum* L.	Subterranean clover	Seaton Park
*Ornithopus compressus* L.	Yellow serradella	Santorini
*Medicago sativa* L.	Lucerne	Aurora

**Table 5:** Phytoestrogens from pasture legume species identified in this study by LC-MS-QToF in positive ionization mode.

Name	Molecular Formula	M + H	Basis for Identification ^a^
**Isoflavones**			
Daidzein	C_15_H_10_O_4_	255.0652	STD
Formononetin	C_16_H_12_O_4_	269.0808	STD
Genistein	C_15_H_10_O_5_	271.0601	STD
**Coumestans**			
Coumestrol	C_15_H_8_O_5_	269.0444	STD
4′-methoxycoumestrol	C_16_H_10_O_5_	283.0579	AM

^a^ Basis for identification codes: AM [43,44]—match to accurate mass/molecular formula and MS/MS spectra; STD—match to accurate mass and retention time of analytical standards.

## Supplementary Materials

The following are available online at https://www.mdpi.com/2218-1989/10/7/267/s1, Figure S1: Hierarchal clustering of molecular features in leaf tissue of annual pasture legumes acquired using UHPLC-QToF-MS in positive and negative mode. Hierarchical clustering algorithm and Euclidean distance metric were used on normalized abundance using MPP (ver. 14.5 Agilent Santa Clara, CA, USA), Figure S2: Hierarchal clustering of relative abundance of flavonoids, their glycosides, and coumestrol in inflorescence tissue in pasture legumes collected in 2016. Hierarchical clustering algorithm and Euclidean distance metric were used on normalized abundance using R package.

## References

[1] Whitbread A.M., Hall C.A., Pengelly B.C. (2009). A novel approach to planting grasslegume pastures in the mixed farming zone of southern inland Queensland, Australia. Crop Pasture Sci..

[2] Hackney B., Dear B., Li G., Rodham C., Tidd J. Current and future use of pasture legumes in central and southern NSW—Results of a farmer and advisor survey. Proceedings of the 14th Australian Society of Agronomy Conference.

[3] Loi A., Howieson J.G., Nutt B.J., Carr S.J. (2005). A second generation of annual pasture legumes and their potential for inclusion in Mediterranean-type farming systems. Aust. J. Exp. Agric..

[4] Burggraaf V., Waghorn G., Woodward S., Thom E. (2008). Effects of condensed tannins in white clover flowers on their digestion in vitro. Anim. Feed Sci. Technol..

[5] Hall D., Wolfe E., Cullis B. (1985). Performance of breeding ewes on lucerne-subterranean clover pastures. Aust. J. Exp. Agric..

[6] Humphries A. (2013). Future applications of lucerne for efficient livestock production in southern Australia. Crop Pasture Sci..

[7] Mulholland J. (1987). Animal production from lucerne-based pastures. Realising the Potential of Pastures, the Proceedings of the 16th Riverina Outlook Conference, Wagga Wagga, Australia, 15 July, 1987.

[8] Loi A., Howieson J.H., Carr S.J. (2001). Register of Australian herbage plant cultivars. *Biserrula pelecinus* L. (biserrula) cv. Casbah. Aust. J. Exp. Agric..

[9] Hackney B., Nutt B., Loi A., Yates R., Quinn J., Piltz J., Jenkins J., Weston L., O’Hare M., Butcher A., Acuña T., Moeller C., Parsons D., Harrison M. (2015). “On-demand” hardseeded pasture legumes–a paradigm shift in crop-pasture rotations for southern Australian mixed farming systems. Building Productive, Diverse and Sustainable Landscapes, Proceedings of the 17th Australian Agronomy Conference, Hobart, Australia, 20–24 September 2015.

[10] Dear B., Ewing M.A. (2008). The search for new pasture plants to achieve more sustainable production systems in southern Australia. Aust. J. Exp. Agric..

[11] Latif S., Gurusinghe S., Weston P.A., Brown W.B., Quinn J.C., Piltz J.W., Weston L.A. (2019). Performance and weed-suppressive potential of selected pasture legumes against annual weeds in south-eastern Australia. Crop Pasture Sci..

[12] Mathesius U. (2018). Flavonoid Functions in Plants and Their Interactions with Other Organisms. Plants.

[13] Weston L.A., Mathesius U. (2013). Flavonoids: Their structure, biosynthesis and role in the rhizosphere, including allelopathy. J. Chem. Ecol..

[14] Hassan S., Mathesius U. (2012). The role of flavonoids in root–rhizosphere signalling: Opportunities and challenges for improving plant–microbe interactions. J. Exp. Bot..

[15] Reed K.F.M. (2016). Fertility of herbivores consuming phytoestrogen-containing *Medicago* and *Trifolium* species. Agriculture.

[16] Tiwari B.K., Brunton N.P., Brennan C. (2013). Handbook of Plant Food Phytochemicals: Sources, Stability and Extraction.

[17] Wocławek-Potocka I., Mannelli C., Boruszewska D., Kowalczyk-Zieba I., Waśniewski T., Skarżyński D.J. (2013). Diverse effects of phytoestrogens on the reproductive performance: Cow as a model. Int. J. Endocrinol..

[18] Hashem N., El-Azrak K., Sallam S. (2016). Hormonal concentrations and reproductive performance of Holstein heifers fed *Trifolium alexandrinum* as a phytoestrogenic roughage. Anim. Reprod. Sci..

[19] Hloucalová P., Skládanka J., Horký P., Klejdus B., Pelikán J., Knotová D. (2016). Determination of Phytoestrogen Content in Fresh-Cut Legume Forage. Animals.

[20] Burton J., Wells M. (2002). The effect of phytoestrogens on the female genital tract. J. Clin. Pathol..

[21] Cvejić J., Bursać M., Atanacković M. (2012). Phytoestrogens: “Estrogene-Like” Phytochemicals. Studies in Natural Products Chemistry.

[22] Bickoff E., Booth A., Lyman R., Livingston A., Thompson C., Deeds F. (1957). Coumestrol, a new estrogen isolated from forage crops. Science.

[23] Mostrom M., Evans T.J. (2011). Phytoestrogens.

[24] Woclawek-Potocka I., Okuda K., Acosta T., Korzekwa A., Pilawski W., Skarzynski D. (2005). Phytoestrogen metabolites are much more active than phytoestrogens themselves in increasing prostaglandin F2α synthesis via prostaglanin F2α synthase-like 2 stimulation in bovine endometrium. Prostaglandins Other Lipid Mediat..

[25] Lookhart G. (1980). Analysis of coumestrol, a plant estrogen, in animal feeds by high-performance liquid chromatography. J. Agric. Food Chem..

[26] Dixon R.A., Achnine L., Kota P., Liu C.J., Reddy M.S., Wang L. (2002). The phenylpropanoid pathway and plant defence—A genomics perspective. Mol. Plant Pathol..

[27] Yu O., Jung W., Shi J., Croes R.A., Fader G.M., McGonigle B., Odell J.T. (2000). Production of the Isoflavones Genistein and Daidzein in Non-Legume Dicot and Monocot Tissues. Plant Physiol..

[28] Gupta O.P., Dahuja A., Sachdev A., Jain P.K., Kumari S., Praveen S. (2019). Cytosine Methylation of Isoflavone Synthase Gene in the Genic Region Positively Regulates Its Expression and Isoflavone Biosynthesis in Soybean Seeds. DNA Cell Biol..

[29] Dixon R.A., Harrison M.J., Paiva N.L. (1995). The isoflavonoid phytoalexin pathway from enzymes to genes to transcription factors. Physiol. Plant..

[30] Piluzza G., Sulas L., Bullitta S. (2014). Tannins in forage plants and their role in animal husbandry and environmental sustainability: A review. Grass Forage Sci..

[31] Salami S.A., Luciano G., O’Grady M.N., Biondi L., Newbold C.J., Kerry J.P., Priolo A. (2019). Sustainability of feeding plant by-products: A review of the implications for ruminant meat production. Anim. Feed Sci. Technol..

[32] Aerts R.J., Barry T.N., McNabb W.C. (1999). Polyphenols and agriculture: Beneficial effects of proanthocyanidins in forages. Agric. Ecosyst. Environ..

[33] Douglas G., Stienezen M., Waghorn G., Foote A., Purchas R. (1999). Effect of condensed tannins in birdsfoot trefoil (*Lotus corniculatus*) and sulla (*Hedysarum coronarium*) on body weight, carcass fat depth, and wool growth of lambs in New Zealand. N. Z. J. Agric. Res..

[34] Ramírez-Restrepo C., Barry T. (2005). Alternative temperate forages containing secondary compounds for improving sustainable productivity in grazing ruminants. Anim. Feed Sci. Technol..

[35] Kingston-Smith A.H., Edwards J.E., Huws S.A., Kim E.J., Abberton M. (2010). Plant-based strategies towards minimising ‘livestock’s long shadow’. Proc. Nutr. Soc..

[36] Tzamaloukas O., Athanasiadou S., Kyriazakis I., Huntley J., Jackson F. (2006). The effect of chicory (*Cichorium intybus*) and sulla (*Hedysarum coronarium*) on larval development and mucosal cell responses of growing lambs challenged with *Teladorsagia circumcincta*. Parasitology.

[37] Hoste H., Torres-Acosta J., Quijada J., Chan-Perez I., Dakheel M., Kommuru D., Mueller-Harvey I., Terrill T. (2016). Interactions between nutrition and infections with *Haemonchus contortus* and related gastrointestinal nematodes in small ruminants. Advances in Parasitology.

[38] Min B.R., Barry T.N., Attwood G.T., McNabb W.C. (2003). The effect of condensed tannins on the nutrition and health of ruminants fed fresh temperate forages: A review. Anim. Feed Sci. Technol..

[39] Mueller-Harvey I., Bee G., Dohme-Meier F., Hoste H., Karonen M., Kölliker R., Lüscher A., Niderkorn V., Pellikaan W.F., Salminen J.-P. (2019). Benefits of condensed tannins in forage legumes fed to ruminants: Importance of structure, concentration, and diet composition. Crop. Sci..

[40] Weston L.A., Skoneczny D., Weston P.A., Weidenhamer J.D. (2015). Metabolic profiling: An overview—New approaches for the detection and functional analysis of biologically active secondary plant products. J. Allelochem. Interact..

[41] Williamson L.N., Bartlett M.G. (2007). Quantitative liquid chromatography/time-of-flight mass spectrometry. Biomed. Chromatogr..

[42] Nehybova T., Smarda J., Benes P. (2014). Plant coumestans: Recent advances and future perspectives in cancer therapy. Anti Cancer Agents Med. Chem..

[43] Christ B., Hauenstein M., Hörtensteiner S. (2016). A liquid chromatography–mass spectrometry platform for the analysis of phyllobilins, the major degradation products of chlorophyll in *Arabidopsis thaliana*. Plant. J..

[44] Jiang H., Liao X., Wood C.M., Xiao C.-W., Feng Y.-L. (2016). A robust analytical method for measurement of phytoestrogens and related metabolites in serum with liquid chromatography tandem mass spectrometry. J. Chromatogr. B.

[45] Smith J., Jagusch K., Brunswick L., Kelly R. (1979). Coumestans in lucerne and ovulation in ewes. N. Z. J. Agric. Res..

[46] Molyneux R.J., Ralphs M.H. (1992). Plant toxins and palatability to herbivores. Rangel. Ecol. Manag. J. Range Manag. Arch..

[47] Oleszek W., Stochmal A., Janda B. (2007). Concentration of isoflavones and other phenolics in the aerial parts of Trifolium species. J. Agric. Food Chem..

[48] Sivesind E., Seguin P. (2005). Effects of the environment, cultivar, maturity, and preservation method on red clover isoflavone concentration. J. Agric. Food Chem..

[49] Mahmood K., Khan M.B., Song Y.Y., Ijaz M., Luo S.M., Zeng R.S. (2013). UV-irradiation enhances rice allelopathic potential in rhizosphere soil. Plant Growth Regul..

[50] Iannucci A., Fragasso M., Platani C., Papa R. (2013). Plant growth and phenolic compounds in the rhizosphere soil of wild oat (*Avena fatua* L.). Front. Plant Sci..

[51] Einhellig F.A. (2018). Allelopathy—A natural protection, allelochemicals. Handbook of Natural Pesticides: Methods.

[52] Visnevschi-Necrasov T., Barreira J.C., Cunha S.C., Pereira G., Nunes E., Oliveira M.B.P. (2015). Phylogenetic insights on the isoflavone profile variations in *Fabaceae* spp.: Assessment through PCA and LDA. Food Res. Int..

[53] Butkutė B., Padarauskas A., Cesevičienė J., Taujenis L., Norkevičienė E. (2018). Phytochemical composition of temperate perennial legumes. Crop. Pasture Sci..

[54] Tsao R., Papadopoulos Y., Yang R., Young J.C., McRae K. (2006). Isoflavone profiles of red clovers and their distribution in different parts harvested at different growing stages. J. Agric. Food Chem..

[55] Wu Q., Wang M., Simon J.E. (2003). Determination of isoflavones in red clover and related species by high-performance liquid chromatography combined with ultraviolet and mass spectrometric detection. J. Chromatogr..

[56] Butkutė B., Lemežienė N., Dabkevičienė G., Jakštas V., Vilčinskas E., Janulis V. (2014). Source of variation of isoflavone concentrations in perennial clover species. Pharmacogn. Mag..

[57] Zgórka G. (2009). Ultrasound-assisted solid-phase extraction coupled with photodiode-array and fluorescence detection for chemotaxonomy of isoflavone phytoestrogens in *Trifolium* L. (Clover) species. J. Sep. Sci..

[58] Ramos G.P., Dias P.M., Morais C.B., Fröehlich P.E., Dall’Agnol M., Zuanazzi J.A. (2008). LC determination of four isoflavone aglycones in red clover (*Trifolium pratense* L.). Chromatographia.

[59] Saviranta N.M., Anttonen M.J., von Wright A., Karjalainen R.O. (2008). Red clover (*Trifolium pratense* L.) isoflavones: Determination of concentrations by plant stage, flower colour, plant part and cultivar. J. Sci. Food Agric..

[60] Bennetau-Pelissero C. (2016). Risks and benefits of phytoestrogens: Where are we now?. Curr. Opin. Clin. Nutr. Metab. Care.

[61] Dubery I.A., Mienie C. (2001). Tissue-specific expression of the chalcone synthase multigene family in *Phaseolus vulgaris*: Development of a RT-PCR method for the expression profiling of the chs isogenes. J. Plant Physiol..

[62] Martin C. (1993). Structure, function, and regulation of the chalcone synthase. International Review of Cytology.

[63] Jung W., Yu O., Lau S.-M.C., O’Keefe D.P., Odell J., Fader G., McGonigle B. (2000). Identification and expression of isoflavone synthase, the key enzyme for biosynthesis of isoflavones in legumes. Nat. Biotechnol..

[64] Dixon R.A., Liu C., Jun J.H. (2013). Metabolic engineering of anthocyanins and condensed tannins in plants. Curr. Opin. Biotechnol..

[65] McMurray C.H., Laidlaw A.S., McElroy M. (1986). The effect of plant development and environment on formononetin concentration in red clover (*Trifolium pratense* L.). J. Sci. Food Agric..

[66] Kim H.-K., Jang Y.-H., Baek I.-S., Lee J.-H., Park M.J., Chung Y.-S., Chung J.-I., Kim J.-K. (2005). Polymorphism and expression of isoflavone synthase genes from soybean cultivars. Mol. Cells.

[67] Jonker A., Peiqiang Y. (2016). The Role of Proanthocyanidins Complex in Structure and Nutrition Interaction in Alfalfa Forage. Int. J. Mol. Sci..

[68] Wang Y., McAllister T.A., Acharya S. (2015). Condensed tannins in sainfoin: Composition, concentration, and effects on nutritive and feeding value of sainfoin forage. Crop. Sci..

[69] Azuhnwi B.N., Boller B., Dohme-Meier F., Hess H.D., Kreuzer M., Stringano E., Mueller-Harvey I. (2013). Exploring variation in proanthocyanidin composition and content of sainfoin (*Onobrychis viciifolia*). J. Sci. Food Agric..

[70] Terrill T.H., Rowan A.M., Douglas G.B., Barry T.N. (1992). Determination of extractable and bound condensed tannin concentrations in forage plants, protein concentrate meals and cereal grains. J. Sci. Food Agric..

[71] Jackson F.S., McNabb W.C., Barry T.N., Foo Y.L., Peters J.S. (1996). The Condensed Tannin Content of a Range of Subtropical and Temperate Forages and the Reactivity of Condensed Tannin with Ribulose- 1,5-bis-phosphate Carboxylase (Rubisco) Protein. J. Sci. Food Agric..

[72] Johnson M.T., Smith S.D., Rausher M.D. (2009). Plant sex and the evolution of plant defenses against herbivores. Proc. Natl. Acad. Sci. USA.

[73] Sumner L., Paiva N., Dixon R., Geno P. (1996). HPLC-Continuous-flow liquid secondary ion mass spectrometry of flavonoid glycosides in leguminous plant extracts. J. Mass Spectrom..

[74] Neilson E.H., Goodger J.Q.D., Woodrow I.E., Møller B.L. (2013). Plant chemical defense: At what cost?. Trends Plant Sci..

[75] Latif S., Gurusinghe S., Weston P.A., Quinn J.C., Piltz J.W., Weston L.A. (2019). Metabolomic approaches for the identification of flavonoids associated with weed suppression in selected Hardseeded annual pasture legumes. Plant Soil.

[76] Dedio W., Clark K. (1968). Biochanin A and formononetin content in red clover varieties at several maturity stages. Can. J. Plant Sci..

[77] Zhou Z., Chen X., Zhang M., Blanchard C. (2014). Phenolics, flavonoids, proanthocyanidin and antioxidant activity of brown rice with different pericarp colors following storage. J. Stored Prod. Res..

[78] Qiu Y., Liu Q., Beta T. (2010). Antioxidant properties of commercial wild rice and analysis of soluble and insoluble phenolic acids. Food Chem..

[79] Min B., McClung A.M., Chen M.H. (2011). Phytochemicals and antioxidant capacities in rice brans of different color. J. Food Sci..

[80] Sun B., Ricardo-da-Silva J.M., Spranger I. (1998). Critical factors of vanillin assay for catechins and proanthocyanidins. J. Agric. Food Chem..

