# Supplementary material for: Metabolic Profiling Provides Unique Insights to Accumulation and Biosynthesis of Key Secondary Metabolites in Annual Pasture Legumes of Mediterranean Origin
Source: Metabolites. 2020 Jun 28;10(7):267. doi: 10.3390/metabo10070267 (PMC7407162; doi:10.3390/metabo10070267)
Supplement: Supplementary file 1 [file metabolites-10-00267-s001.pdf]

**Supplementary Data**

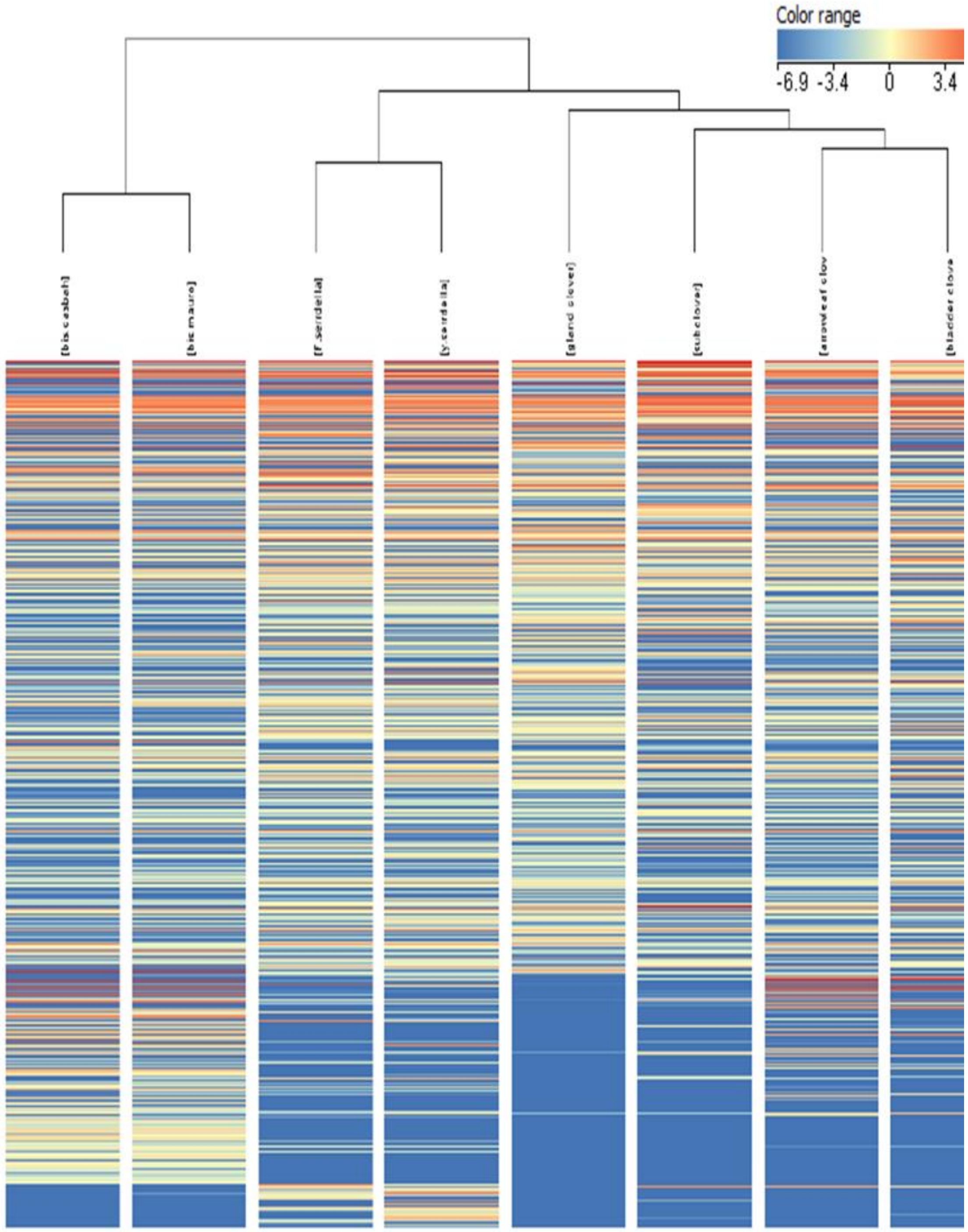


**Figure S1.** Hierarchal clustering of molecular features in leaf tissue of annual pasture legumes acquired using UHPLC-QTOF-MS in positive and negative mode. Hierarchical clustering algorithm and Euclidean distance metric were used on normalized abundance using MPP (ver. 14.5 Agilent Santa Clara, CA, USA).

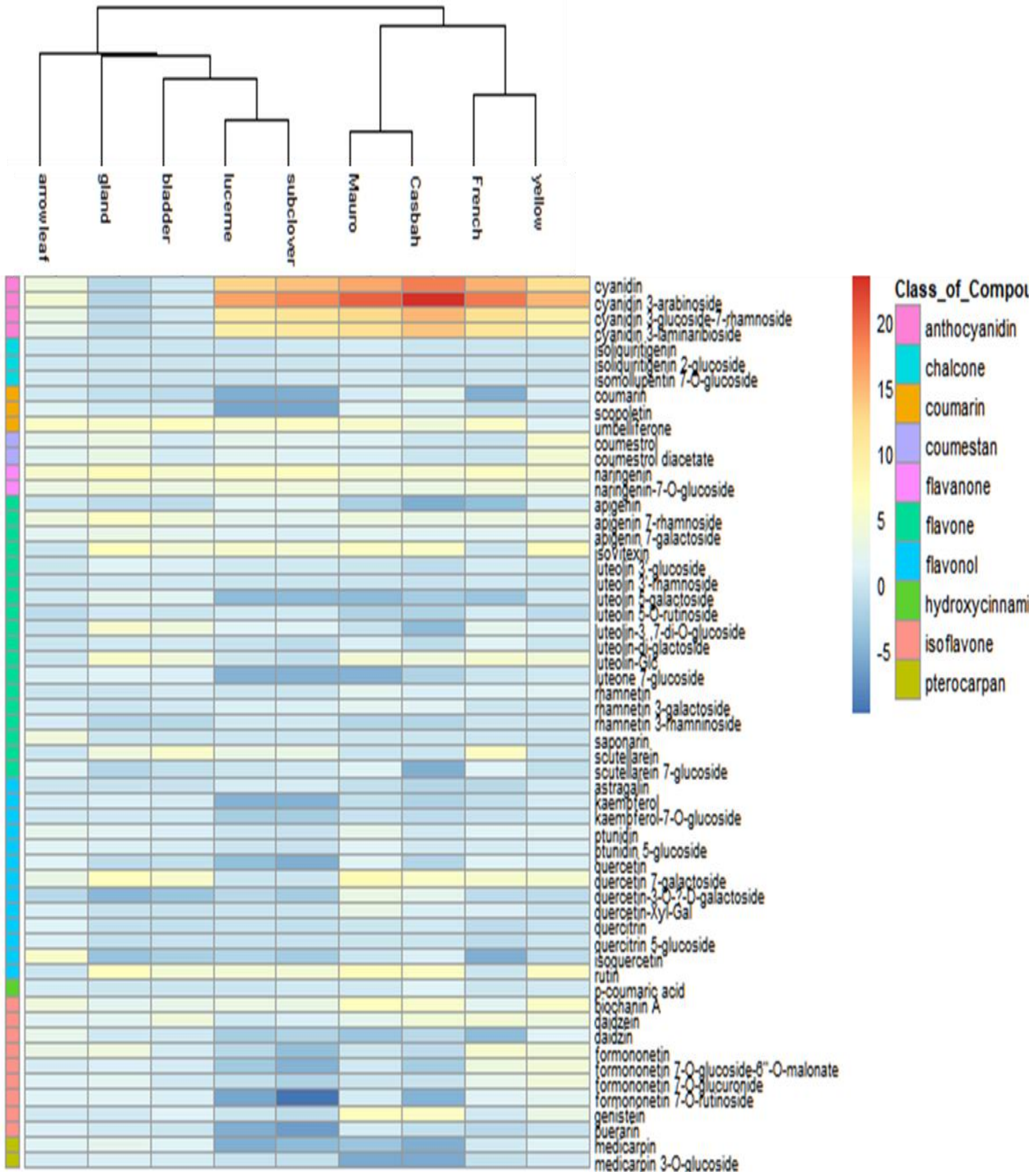


Figure S2. Hierarchal clustering of relative abundance of flavonoids, their glycosides and coumestrol in inflorescence tissue in pasture legumes collected in 2016. Hierarchical clustering algorithm and Euclidean distance metric were used on normalized abundance using R package.
